# LncRNA MEG3 suppressed the progression of ovarian cancer via sponging miR-30e-3p and regulating LAMA4 expression

**DOI:** 10.1186/s12935-020-01259-y

**Published:** 2020-05-24

**Authors:** Yang Liu, Yangchun Xu, Lei Ding, Lili Yu, Butian Zhang, Dan Wei

**Affiliations:** 1 Department of Radiation Oncology, Affiliated Cancer Hospital of Zhengzhou University, Henan Cancer Hospital, Zhengzhou, 450008 Henan China; 2 Department of Dermatology, The Second Hospital of Jilin University, Changchun, 130062 Jilin China; 3 Department of Radiology, China-Japan Union Hospital of Jilin University, Changchun, 130028 Jilin China; 4 Key Laboratory of Birth Defect Prevention of National Health Commission, Zhengzhou, 450002 Henan China; 5 School of Life Sciences and Technology, Xinxiang Medical University, No. 601 Jinsui Avenue, Hongqi District, Xinxiang, 453003 Henan China

**Keywords:** LncRNA MEG3, LAMA4, miR-30e-3p, Ovarian cancer

## Abstract

**Background:**

Ovarian cancer (OC) is a common female reproductive malignancy with a high mortality rate. Although LAMA4 was observed to be downregulated in OC cells, its mechanism in regulating OC metastasis is still unknown. This study aimed to investigate the effect of LAMA4 and its mechanism on OC.

**Methods:**

To achieve this aim, a microarray analysis was performed to screen out the key genes involved in OC pathogenesis. Western-blot and qRT-PCR assays were also carried out to detect protein and mRNA expressions, respectively. A luciferase reporter assay was further used to confirm the direct interaction of miR-30e-3p with MEG3, and the direct interaction of miR-30e-3p with LAMA4 mRNA. Cytological experiments (CCK8, colony formation assay, wound-healing assay etc.) were then performed to explore the roles of miR-30e-3p, MEG3, and LAMA4 in OC cells.

**Results:**

After carrying out microarray analysis, LAMA4 was confirmed as a key gene associated with OC pathogenesis. Research results proved that miR-30e-3p was markedly upregulated, while MEG3 and LAMA4 were noticeably downregulated in OC tissues and cells. The overexpression of LAMA4 significantly impaired the proliferation, migration, and invasion of OC cells. However, the upregulation of MEG3 increased the expression of LAMA4 by sponging miR-30e-3p, which alleviated the malignancy of OC cells.

**Conclusions:**

Observations showed that forced LAMA4 overexpression could inhibit OC progression, which was regulated by MEG3 via sponging miR-30e-3p. The findings of this research could provide new insights into the mechanism by which MEG3 and LAMA4 exert their anti-oncogenic roles in OC progression.

*Trial registration* Not applicable

## Background

Ovarian cancer (OC) refers to a malignancy commonly associated with female residents, and health statistics indicate that it a formidable threat to women’s well-being [1]. In a study in which patients suffering from OC were diagnosed at terminal stages, the 5-year overall survival rate of the OC patients was less than 30%. While more efficient treatment methods for OC patients have been developed in recent years, it is pertinent to develop a comprehensive study and understand the molecular mechanism contributing to OC progression.

Long non-coding RNAs (LncRNAs) are crucial to OC treatments. They have been found to act as the key roles in immune response, cancer progression, cell growth and cell differentiation [2–6]. Accumulating evidence has also revealed that by sponging, lncRNAs could suppress the activities of miRNAs to derepress the targets of miRNAs at the posttranscriptional level [7]. For instance, LINC01234 contributed to the tumorigenesis of gastric cancer in vivo via sponging miR-204-5p to regulate CBFB expression. In another research on OC, lncRNA NEAT1 overexpression was proved to be correlated with cell growth and migration via sponging miR-506 [8]. The maternally expressed gene 3 is located on chromosome 14q32, which can encode a lncRNA-MEG3 (MEG3) [9]. This substance has been thought to be a tumor suppressor in various cancers [10–12]. In another study on OC, MEG3 acted as the antitumor role by promoting cell cycle arrest and inhibiting cell proliferation. Although ample studies have noted the significance of MEG3 in OC, its OC development mechanism involved in the miRNA–mRNA network is still not fully clear.

Located in mesenchyme-derived tissues, laminin alpha4 (LAMA4) is widely distributed in adult human tissues [13]. Several studies found that the abnormal expression of LAMA4 was related to the formation and function of the endothelium, including the cell migration and invasion of certain types of cancers in vivo and in vitro [14, 15]. For example, the LAMA4-knockdown impaired the cell invasion capability in gastric cancer by inhibiting MMP2 expression [16]. In vivo, LAMA4 with high expression was observed in human hepatocellular carcinoma tissues (n = 48) compared with the corresponding adjacent tissues, and the upregulation of LAMA4 was closely associated with tumor invasion and metastasis [17]. Besides, the LAMA4 expression was thought to be the symbol of human pre-malignant lesions to malignant carcinomas in breast cancer. The effect and mechanism of LAMA4 on OC, however, are yet to be fully understood.

The present study aimed to explore the effect of LAMA4 and its mechanism on OC. The qRT-PCR, western blot, and cytological experiments (e.g., CCK8 and transwell assay) were used to prove that the mechanism of LAMA4 in regulating OC involved in the lnc-MEG3/miR-30e-3p axis.

## Materials and methods

### Microarray analysis

The GEO series of GSE29450 and GSE54388 were downloaded from GEO DataSets (http://www.ncbi.nlm.nih.gov/gds). GSE2940 data included 10 normal ovarian tissue specimens and 10 OC tissue specimens. The GSE54388 data were comprised of 6 normal ovarian tissue specimens and 16 OC tissue specimens. A GEO2R algorithm was used to identify differentially expressed genes (DEGs) of the two GSE data series. The three functional enrichment analysis software applications were used to analyze the common signaling pathway based on the KEGG enrichment for 2160 common DEGs, such as WebGestalt (http://www.webgestalt.org/option.php), Metascape (http://metascape.org/) and KOBAS (http://kobas.cbi.pku.edu.cn/index.php). A public online database named String (http://string-db.org/) was also utilized to further construct the co-expression network and analyze the Gene Ontology (GO) enrichment and KEGG enrichment of the common DEGs involved in the same pathway in the three functional enrichment analysis software applications. The RNA-Seq, which was obtained from an online cancer database (TCGA), was also used to separate the genes associated with OC pathogenesis. With the Kaplan–Meier plotter (http://kmplot.com/analysis/), a survival analysis was performed to assess the effect of the screened genes on the prognosis of OC. Finally, the differential expression of the screened gene was analyzed with GEPIA (http://gepia.cancer-pku.cn/index.html), an online web for evaluating the RNA-Seq from TCGA.

### Clinical ovarian tissue samples

With the informed consent of patients, 40 tissue specimens (30 OC tissues and 10 normal ovarian tissues) were collected from Xinxiang Medical University. After assessing the ethical implications of the research, the Ethics Committee of the Xinxiang Medical University approved this study. We ensured that the chosen patients had not gone through radiotherapy or chemotherapy prior to surgery. The patients’ detailed clinical baseline data include age, tumor stage, differentiation, and omentum metastasis (Table 1).

**Table 1:** Baseline characteristics of the 30 patients of ovarian cancer

Characteristics	Value
Age-year	
Mean	51
Range	30–76
> 50	16 (53.3%)
≤ 50	14 (46.7%)
Tumor stage-no. (%)	
I or II	18 (60%)
III or IV	12 (40%)
Differentiation-no. (%)	
Well differentiated	17 (56.7%)
Poorly differentiated	13 (43.3%)
Omentum metastasis-no. (%)	
Absent	14 (46.7%)
Present	16 (53.3%)
Missing data	1 (3.3%)

### Cell line acquisition and cell culture

Human OC cell lines (SKOV3, OVCAR3, and Caov-4), human normal ovarian epithelial cell line (IOSE80), and 293T cell line were obtained from ATCC (USA). Another human healthy ovarian cell line (HOSEpiC) was purchased from ScienCell Research Laboratories (Cat#: 7310, USA). SKOV3 cells were cultured in McCoy’s 5A medium (Gibco, USA) with 10% (v/v) fetal bovine serum (FBS, Invitrogen, USA). Caov-4, IOSE-80, and 293T cell lines were cultured in DMEM medium (HyClone, USA) and supplemented with 10% (v/v) FBS, 100 U/mL penicillin, and 100 μg/mL streptomycin. HOSEpiC cell line and OVCAR3 cell line were cultured in RPMI-1640 medium (HyClone, USA) and augmented with 10% (v/v) FBS, 100 U/mL penicillin, and 100 μg/mL streptomycin. All the cells were placed in an atmosphere of 5% CO_2_ at 37 °C.

### Cell transfection

For cell transfection, the miR-30e-3p mimic and the miRNA negative control (NC) were designed and purchased from RiboBio (China). The LAMA4 and MEG3 sequences were inserted into the pcDNA3.1 vector (Invitrogen, USA) to construct the overexpression plasmids of LAMA4 and MEG3. The MEG3 or LAMA4 3′UTR wild-type fragment, which contained the binding sites of miR-30e-3p or its mutant fragment, was synthesized by PCR and cloned into pGL3 plasmids (Promega Corporation, USA) containing the firefly luciferase gene, which was named as pGL3-MEG3-Wt, pGL3-MEG3-MU, pGL3-LAMA4 3′UTR-Wt or pGL3-LAMA4 3′UTR-MU. Then, the SKOV3, OVCAR3, and 293T cells were seeded and cultured overnight before transfection. Lipofectamine 2000 (Thermo Fisher Scientific, USA) was also used to transfect cells based on the manufacturer’s instructions. The nomenclature of the cells co-transfected with MEG3 overexpression and miR-30e-3p mimic was p-MEG3/miR-30e-3p cells, while that of the cells transfected with MEG3 overexpression was p-MEG3/− cells. The control group was regarded as the blank group, while the NC group was considered as the negative control group.

### The detection of mRNA expressions

TRIzol Reagent (Invitrogen, USA) was used to isolate the total RNAs from 40 OC tissue specimens and five cell-line specimens. The concentration and purity of the RNAs were assessed with a UV spectrophotometer. With the aid of PrimeScript RT Kits (TaKaRa, China), 1 μg of extractive RNA was used to synthesize the cDNA. qRT-PCR was carried out using SYBR Premix Ex Taq (TaKaRa, China) on the Real-Time PCR System (Applied Biosystems 7500, USA) to detect the mRNA expression. With the 2^−ΔΔCT^ method, the mRNA relative expressions of LAMA4, MEG3, and miR-30e-3p were calculated. GAPDH, which served as the internal control, was used to normalize the expressions of lncRNA and mRNA, and U6 was used for the miRNA. The primers for qRT-PCR are illustrated in Table 2.

**Table 2:** The list of primer sequences for qRT-PCR

	Sequence (5′-3′)
LAMA4-F	5′-ATGCCGTACTCTGCTGGTTG-3′
LAMA4-R	5′-CTCTCCTGTTGTGTTCCGCT-3′
MEG3-F	5′-GGAGCTGTTGAGCCTTCAGT-3′
MEG3-R	5′-ATTGAGAGCACAGTGGGGTG-3′
miR-30e-3p-F	5′-GGGCTTTCAGTCGGATGTTTACAGC-3′
miR-30e-3p-R	5′-CAGTGCGTGTCGTGGAGT-3′
GAPDH-F	5′-GTCTCCTCTGACTTCAACAGCG-3′
GAPDH-R	5′-ACCACCCTGTTGCTGTAGCCAA-3′
U6-F	5′-CTCGCTTCGGCAGCACATATACT-3′
U6-R	5′-ACGCTTCACGAATTTGCGTGTC-3′

### The detection of protein levels using western blot assay

Protein samples were extracted from cells, and the concentration of each sample was noted. The samples were then separated by 8% SDS-PAGE gels and electrically transferred onto PVDF membranes. After the transfer process, 5% fat-free milk was used to block the membranes for 120 min. The membranes were then incubated overnight at 4 °C with the primary antibodies against LAMA4 (Cat#: ab242359, Abcam, UK) and β-actin (Cat#: ab179467, Abcam, UK). The next day, the membranes were incubated with corresponding secondary antibodies conjugated with HRP (1:5000, Cat#: ab6789, Abcam, UK) for 120 min. The antigen–antibody complexes were visualized using the chemiluminescence detection reagent (Millipore Corporation, USA). The band intensities were read with the FluorChem FC2 Western Blot Imager (AlphaInnotech, San Leandro, CA, USA).

### Cell proliferation assessment

Cell Counting Kit-8 (CCK-8), purchased from KeyGen BioTECH (China), was used to assess cell proliferation. The pretreated SKOV3 or OVCAR3 cells (2.0 × 10^4^ cells/well) were seeded in 96-well plates with 100 μL of complete medium (containing 10% FBS) and were cultured for 0 h, 24 h, 48 h or 72 h. Added to each well at the time of harvest was 10 μL of CCK-8 reagent. After a 2-h incubation period, the absorbance at 450 nm was measured with a microplate reader to assess the cell-proliferation ability. In addition, the colony formation assay was also conducted to evaluate cell proliferation. Pretreated SKOV3 or OVCAR3 cells (600 cells/well) were seeded into 6-well plates, cultured in complete medium, and incubated for 2 weeks. The colonies were fixed in paraform, washed by PBS, stained by 0.1% crystal violet, and observed with the aid of a microscope. The number of cell colonies was counted using a microscope, with colonies more than 50 cells.

### Cell migration assessment

A wound-healing assay is a prominently used technique for investigating the ability of cell migration. Pretreated SKOV3 and OVCAR3 cells (2 × 10^5^ cells/well) were seeded into 6-well plates and cultured until 80% confluence was achieved. A sterile plastic tip was used to scratch the cell layer gently. After washing away the loose cells, the serum-free medium was added to each well and cultured for 0 h or 48 h. With an inverted microscope, the ability of cell migration was assessed by measuring the wound width from four random fields (×100). The migration rate was calculated as (wound width at 0 h − wound width at 48 h)/wound width at 0 h × 100%. The wound widths of different groups at the same time point were measured with the same magnification.

### Cell invasion assessment

The ability of cell invasion was evaluated using the transwell invasion assay. The pretreated SKOV3 and OVCAR3 cells were resuspended in, 200 µL of FBS-free medium. The cells at the density of 1 × 10^5^ cells/well were then plated onto the upper chambers with 8.0-μm pore membranes coated with matrigel. The 600 µL of complete medium (containing 10% FBS) was added to the bottom chamber. After a 2-day incubation period, 3.8% formaldehyde was used for 20 min to fix the invaded cells adhered to the lower side of the membrane. The cells were later stained with 0.1% crystal violet. The numbers of invaded cells on the membranes were photographed with a phase-contrast microscope (Nikon, Japan), and the numbers of invaded cells were calculated from six randomly selected fields (×100).

### Live imaging of OC xenograft in mice models

Two groups of SPF-grade mice (4 weeks old, n = 4 per group) were subcutaneously injected with 1 × 10^6^ SKOV3 cells that were stably transfected with negative control plasmids or LAMA4 overexpression plasmids and kept for 2 months. d-Luciferin potassium salts were injected into the mice before the live imaging of the animals with an IVIS 200 bioluminescence imaging system and Living Image software (Caliper Life Sciences, Hopkinton, MA).

### Luciferase reporter assay

The 293T cells were loaded into 96-well plates to perform the luciferase reporter assay. When the confluence was over 60%, the cells were co-transfected with 400 ng of the constructed plasmids described above (pGL3-MEG3-Wt, pGL3-MEG3-Mut, pGL3-LAMA4 3′UTR-Wt or pGL3-LAMA4 3′UTR-MU), 50 ng of a renilla luciferase reporter vector (pRL-TK), and 50 nM of miR-30e-3p mimic or NC. The Dual-Luciferase Reporter Assay Kit purchased from Promega (USA) was leveraged to measure the relative luciferase activity. The activities of renilla luciferase were later used to normalize the firefly luciferase activity.

### Statistical analysis

SPSS 18.0 software was used to analyze the data collected from the experiments. All the data in our study were presented as mean ± standard deviation. The statistical comparisons between two groups were evaluated using Student’s t-test, and those between multiple groups were evaluated using one-way ANOVA. The statistical significance was considered when *P *< 0.05.

## Results

### LAMA4 was found to be closely related to OC and significantly downregulated in OC

**Fig. 1:**
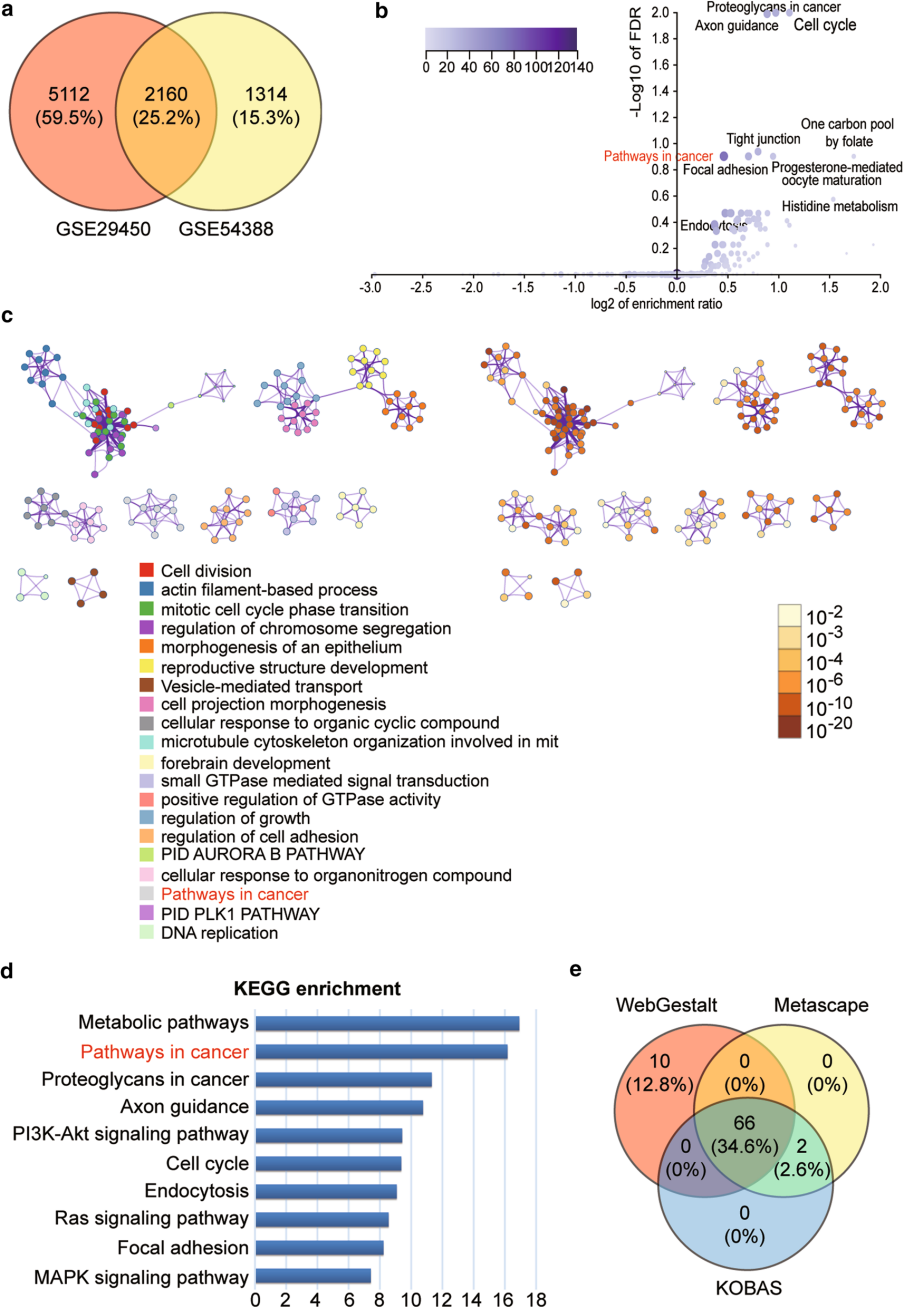
Identification of potential genes of interest that participate in ovarian cancer using a bioinformatics method. **a** A Venn diagram showing the common 2160 DEGs from GSE29450 and GSE54388 data series downloaded from GEO database. **b** The KEGG enrichment result for the 2160 common DEGs analyzed by WebGestalt. **c** The KEGG enrichment result of 2160 common DEGs using Metascape. **d** KOBAS was employed to conduct the KEGG enrichment of the 2160 common DEGs. **e** Genes involved in ‘pathways in cancer’ from WebGestalt, Metascape, and KOBAS algorithms

**Fig. 2:**
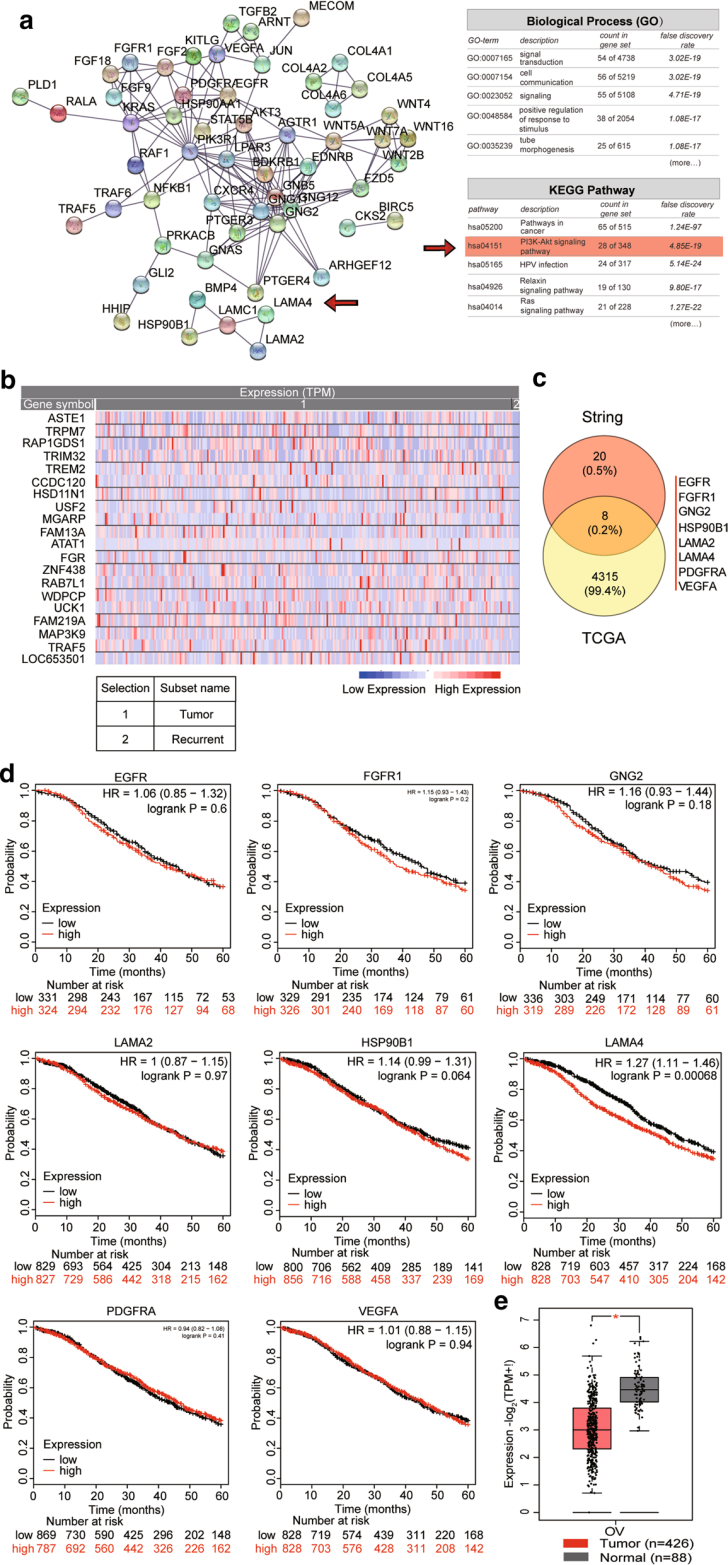
LAMA4 was confirmed as a key and interesting gene in ovarian cancer. **a** The networking relationship between the identified 66 genes was constructed using String. The enriched biological processes and KEGG pathways were illustrated. The selected term was marked using red arrows. **b** The top 20 DEGs from TCGA RNA-Seq were exhibited involving in ovarian cancer. **c** The intersection between the PI3K–Akt signaling-related genes and the DEGs from RNA-seq data from TCGA. Eight genes were identified. **d** The prognostic values of the eight genes in ovarian cancer were analyzed using Kaplan–Meier Plotter. **e** GEPIA data analysis exhibited the differential expression of LAMA4 in ovarian cancer compared with the normal ovary

**Fig. 3:**
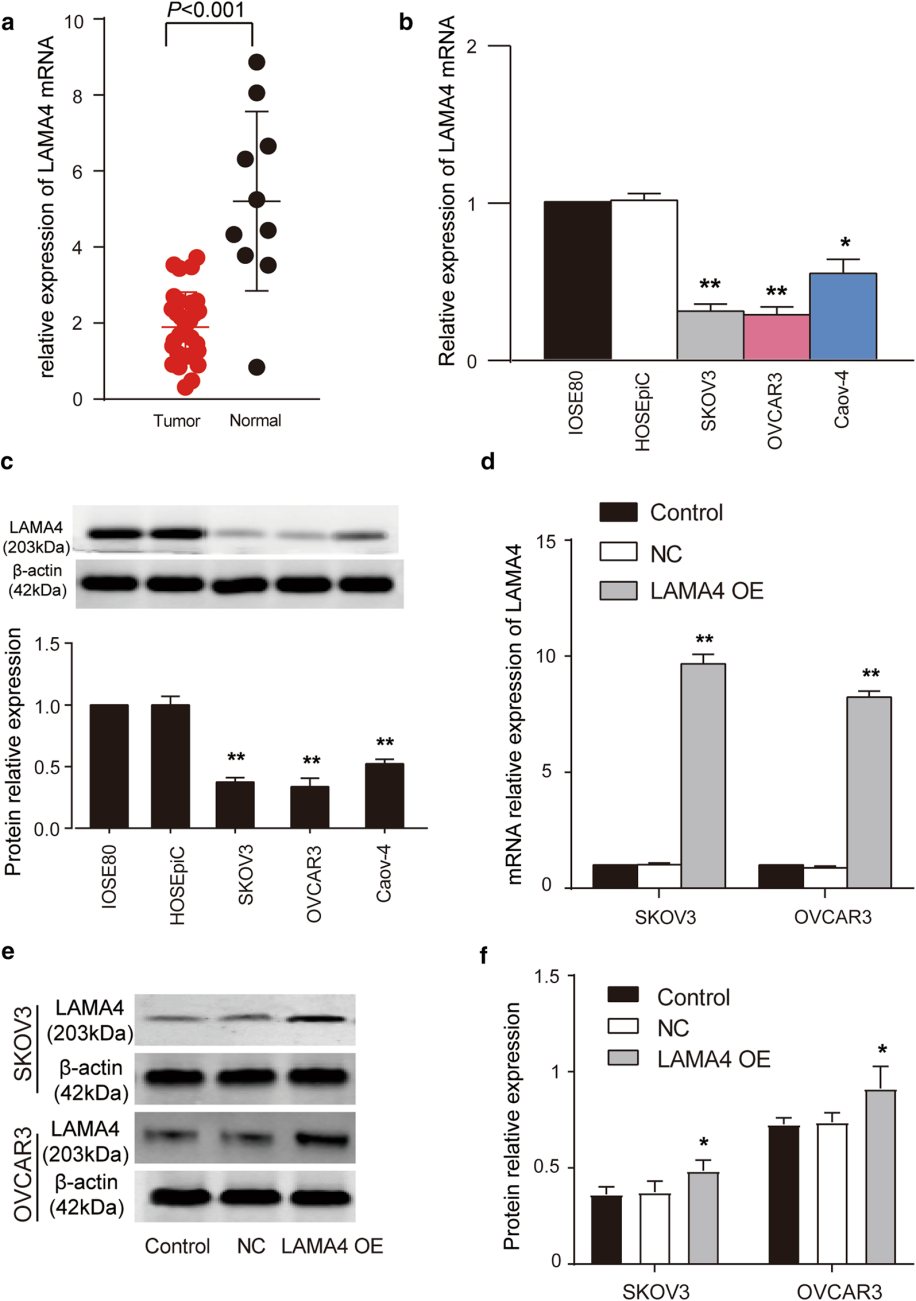
The expressions of LAMA4 in ovarian cancer tissues and cell lines. **a** The mRNA level of LAMA4 was detected by qRT-PCR in healthy and tumorous ovarian tissues. **b** The mRNA expression of LAMA4 in immortalized healthy ovarian cell lines (IOSE80 and HOSEpiC) and OC cell lines (SKOV3, OVCAR3, and Caov-4). *P < 0.05, **P < 0.01 vs. IOSE80. **c** The western-blot assay was used to measure the protein level of LAMA4 in healthy ovarian cell lines (IOSE80 and HOSEpiC) and ovarian cancer cell lines (SKOV3, OVCAR3, and Caov-4). **P < 0.01 vs. IOSE80. **d** The qRT-PCR results of control, NC and LAMA4 OE groups in SKOV3 and OVCAR3 cell lines. **P < 0.01 vs. control group. LAMA4 over-expression was constructed using the pcDNA3.1 vector. Control group is the blank group. NC, Negative control, the cells were transfected with empty plasmids. LAMA4 OE, LAMA4 overexpression, the cells were transfected with LAMA4 over-expression plasmids. **e**, **f** The western blot results of control, NC and LAMA4 OE groups in SKOV3 and OVCAR3 cells. LAMA4 over-expression was constructed using the pcDNA3.1 vector. Control group is the blank group. NC, negative control, the cells were transfected with empty plasmids. LAMA4 OE, LAMA4 overexpression, the cells were transfected with LAMA4 over-expression plasmids. *P < 0.05 vs. control

A total of 7272 DEGs and 3474 DEGs was identified from the GSE29450 data series and GSE54388 data series, respectively. A Venn diagram was also drawn to show the identified 2160 common DEGs from GSE29450 and GSE54388 data series (Fig. 1a). The KEGG enrichment was then performed to annotate the 2160 common genes using WebGestalt, Metascape, and KOBAS algorithms (Fig. 1b–d, respectively). Among the enriched terms, we observed that the 2160 DEGs were significantly enriched in ‘pathways in cancer’ according to the results of the three algorithms. We then intersected the genes in ‘pathways in cancer’ from the three algorithms. The results indicated that 66 common genes were screened out to involve in the ‘pathways in cancer’ (Fig. 1e). By uploading the gene symbols of the 66 common genes to String, we found that 28 genes were closely associated with the PI3K-Akt signaling pathway (Fig. 2a), which has been proved to be closely associated with human cancer pathogenesis including OC pathogenesis [18, 19]. We then interrogated the RNA-Seq results of OC from TCGA. A representative expression heatmap showing the DEGs of the TCGA database is indicated in Fig. 2b. As shown in Fig. 2c, eight genes (EGFR, FGFR1, GNG2, HSP90B1, LAMA2, LAMA4, PDGFRA, and VEGFA) out of the 28 genes were found to be PI3K–Akt signaling-associated and significantly differentially expressed in OC. After carrying out a 5-year survival analysis of the 8 genes using the Kaplan–Meier Plotter (http://kmplot.com/analysis/), we found that only LAMA4 was closely related to OC prognosis (Fig. 2c) and that a higher level of LAMA4 demonstrated a poorer survival outcome in OC patients. In addition, a significantly lower level of LAMA4 expression was observed in OC via Gene Expression Profiling Interactive Analysis (GEPIA) (Fig. 2e). Therefore, we focused on the role of LAMA4 in OC. LAMA4 mRNA was found to be significantly downregulated in our collected clinical samples (Fig. 3a) and cell lines (Fig. 3b). LAMA4 mRNA in tumor tissues and cell lines was 50% lower than that in healthy tissues and cell lines. LAMA4 protein expression was also detected in cell lines, and our results showed that LAMA4 protein was significantly downregulated in OC cell lines such as SKOV3, OVCAR3 and Caov-4 cell lines (Fig. 3c). Before studying the effects of LAMA4 in OC, the overexpression efficiency of LAMA4 overexpression plasmids was validated at mRNA and protein levels. LAMA4 overexpression plasmids transfection led to over 6 times of LAMA4 mRNA upregulation (Fig. 3d) and approximately a 30% increase in LAMA4 protein expression (Fig. 3e, f).

### LAMA4 impaired the malignancy phenotypes in vitro and tumorigenesis in vivo

**Fig. 4:**
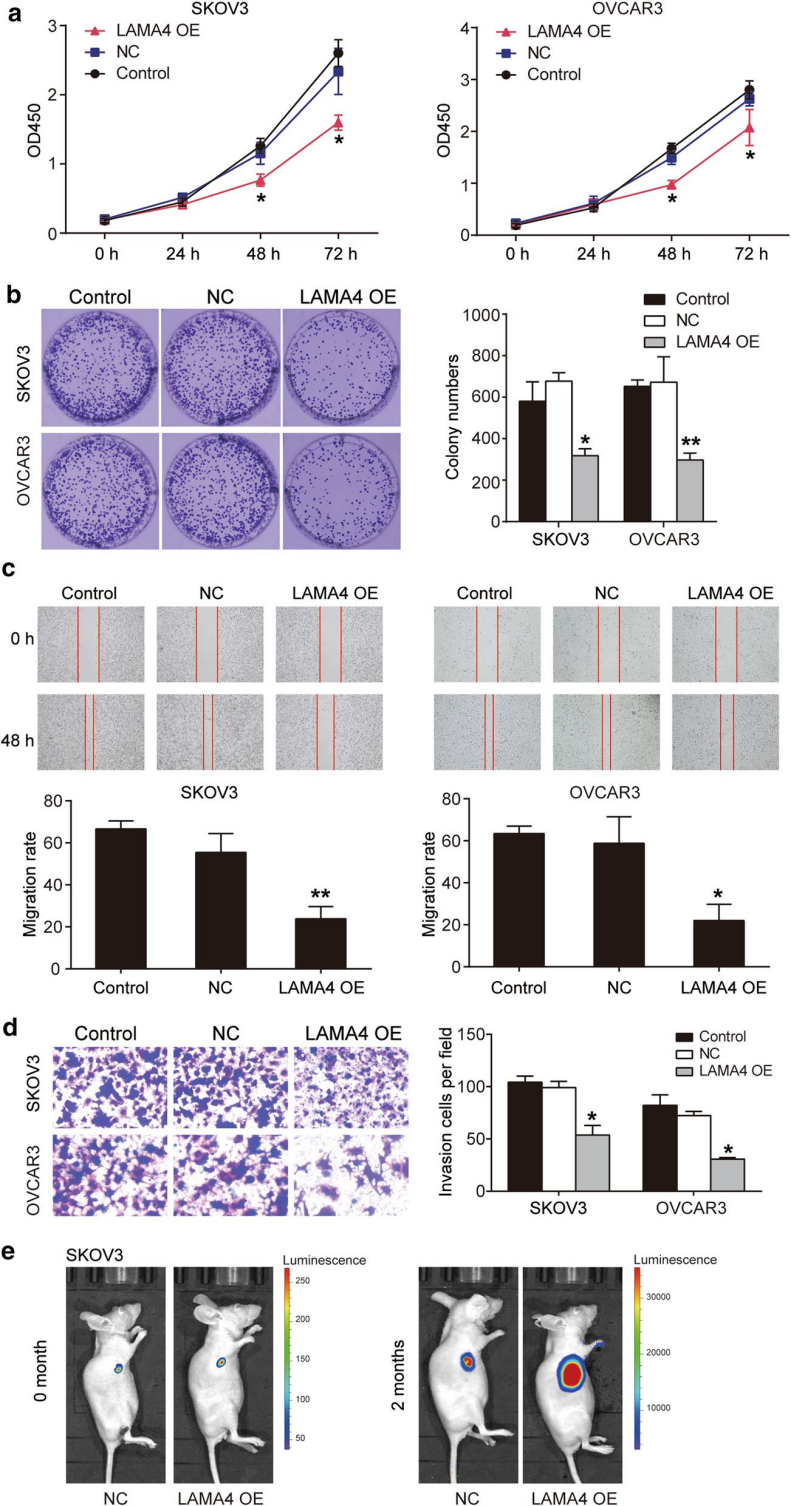
LAMA4 negatively regulated the proliferation, migration, invasion of ovarian cancer cells, and tumorigenesis in animal models. **a** The proliferation of SKOV3 and OVCAR3 cells with LAMA4 over-expression was detected by CCK8 assay. *OD450* optical density at 450 nm. **b** The proliferation of SKOV3 and OVCAR3 cells with LAMA4 over-expression was further determined using colony foci formation assay. **c** The ability of cell migration in SKOV3 and OVCAR3 cells with LAMA4 over-expression was evaluated by wound healing assay. The migration rate was calculated as (wound width at 0 h − wound width at 48 h)/wound width at 0 h ×100%. **d** The ability of cell invasion was assessed in SKOV3 and OVCAR3 cells with LAMA4 over-expression using transwell invasion assay. **e** The bioluminescence results showing the tumorigenesis in vivo. Control group is the blank group. NC, the cells were transfected with empty plasmids. LAMA4 OE, the cells were transfected with LAMA4 over-expression plasmids. *P < 0.05 and **P < 0.01 vs. control

By carrying out cytological experiments, the effects of LAMA4 overexpression on SKOV3 and OVCAR3 cell phenotypes were explored. The CCK-8 assay results showed that the forced expression of LAMA4 resulted in a significant decline (approximately 50% decline) in proliferation of SKOV3 and OVCAR3 cells at 48 h and 72 h (Fig. 4a). The colony-formation assay findings also showed that LAMA4 overexpression markedly inhibited the proliferation of SKOV3 and OVCAR3 cells by approximately 50% (Fig. 4b). The wound-healing assay results showed that after 48 h, the migrated width in the LAMA4 overexpression group reduced by 43% in SKOV3 cells and 18% in OVCAR3 cells, compared with the control group (Fig. 4c). The results of the transwell invasion assays in Fig. 4d showed that LAMA4 overexpression decreased the number of invading cells by 63.8% in SKOV3 and 46.1% in OVCAR3 cells, compared with the control cells. In addition, our in vivo animal assay results demonstrated that the overexpression of LAMA4 in the SKOV3 cell line significantly suppressed tumorigenesis (Fig. 4e). It was also found that LAMA4 overexpression effectively impaired OC progression in vitro and in vivo.

### miR-30e-3p targeted LAMA4 3′UTR, down-regulated LAMA4 expression, and enhanced proliferation and invasion of OC cells

**Fig. 5:**
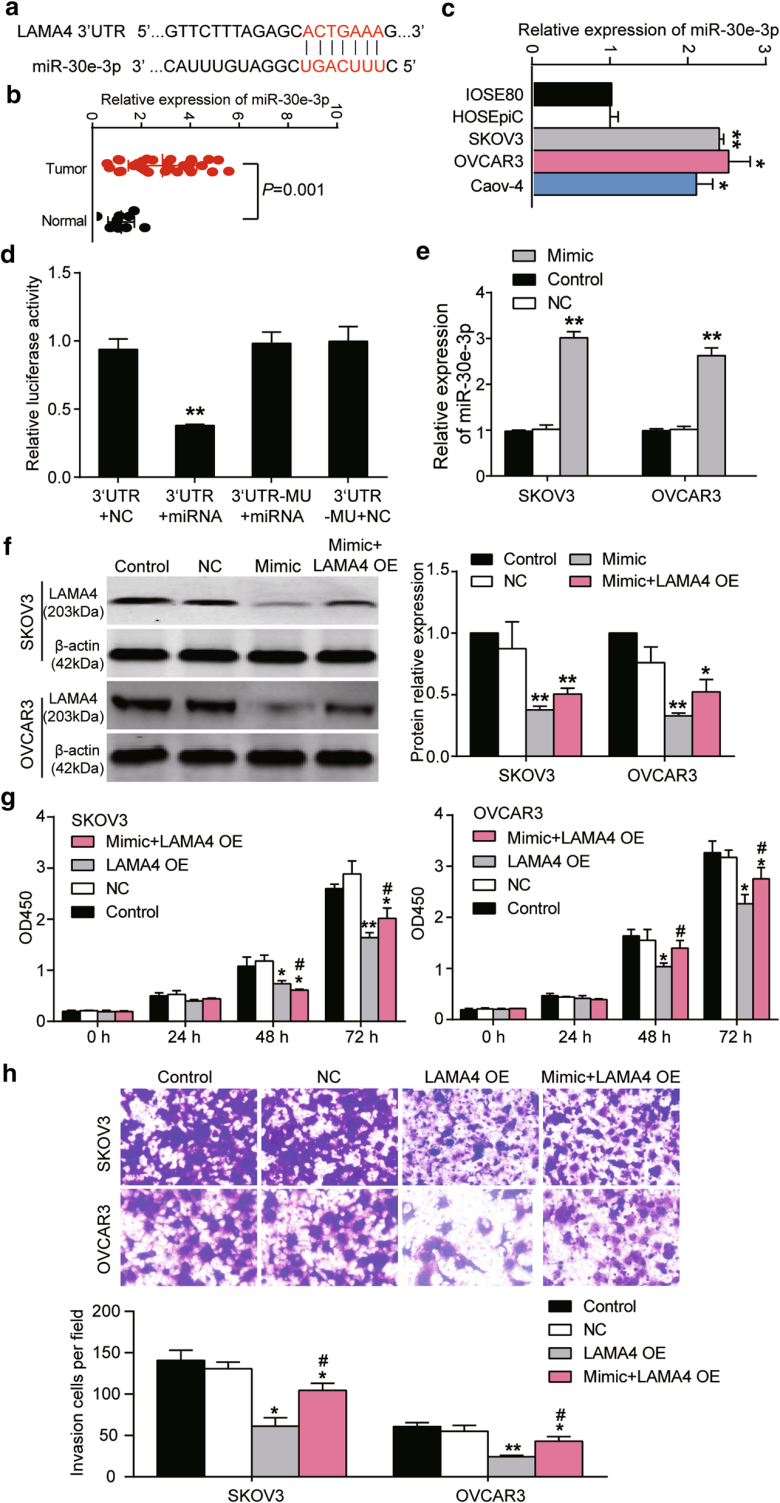
miR-30e-3p reversed the effect of LAMA4 in SKOV3 and OVCAR3 cells. **a** The scheme illustrating the regulatory association between LAMA4 3′UTR and miR-30e-3p. The binding sequences were predicted by miRDB database. **b** The relative expression of miR-30e-3p in healthy and cancerous ovarian tissues. **c** The expression of miR-30e-3p in healthy cell lines IOSE80 and HOSEpiC, and OC cell lines SKOV3, OVCAR3 and Caov-4 cell lines. *P < 0.05, **P < 0.01 vs. IOSE80 cell line. **d** The relative luciferase activities in wild-type and mutated-type LAMA4 3′UTR group co-transfected with miR-30e-3p mimic or miR-30e-3p NC. The experiments were conducted in 293T cells. 3′UTR + NC, the cells were transfected with pGL3-LAMA4 3′UTR-Wt; 3′UTR + miRNA, the cells were co-transfected with pGL3-LAMA4 3′UTR-Wt and miR-30e-3p mimic. 3′UTR-MU + miRNA, the cells were co-transfected with pGL3-LAMA4 3′UTR-Mut and miR-30e-3p mimic. 3′UTR-MU + NC, the cells were transfected with pGL3-LAMA4 3′UTR-Mut. **P < 0.01 vs. 3′UTR + NC. **e** qRT-PCR analysis of the levels of miR-30e-3p in SKOV3 and OVCAR3 cells transfected with miR-30e-3p mimic. **P < 0.01 vs. control group. **f** Western blot analysis of the expression of LAMA4 protein in SKOV3 and OVCAR3 cell lines transfected with miR-30e-3p mimic with or without LAMA4 over-expression. *P < 0.05, **P < 0.01 vs. control group. **g** The proliferation of SKOV3 and OVCAR3 cells transfected with LAMA4 over-expression plasmids alone or co-transfected with miR-30e-3p mimic was detected by CCK8 assay. *P < 0.05, **P < 0.01 vs. control group, ^#^P < 0.05 vs. LAMA4 OE group. **h** The invasion of SKOV3 and OVCAR3 cells transfected with LAMA4 over-expression plasmids alone or co-transfected with miR-30e-3p mimic was detected by Transwell invasion assay. *P < 0.05, **P < 0.01 vs. control group, ^#^P < 0.05 vs. LAMA4 OE group. Control group is the blank control. NC, the cells were transfected with empty plasmids. Mimic, the cells were transfected with miR-30e-3p mimic. Mimic + LAMA4 OE, the cells were co-transfected with miR-30e-3p mimic and LAMA4 over-expression plasmids

The potential target sites of miR-30e-3p on LAMA4 3′UTR were predicted by miRDB. These sites are illustrated in Fig. 5a. miR-30e-3p was significantly upregulated in human OC tissues (Fig. 5b) and OC cell lines SKOV3, OVCAR3 and Caov-4 (Fig. 5c). The expression level of miR-30e-3p in tumor tissues and cell lines was more than two times of that in healthy tissues and cell lines. To confirm the regulatory binding relationship between MEG3 and miR-30e-3p, we performed a dual-luciferase reporter gene assay. The results revealed that miR-30e-3p directly targeted the 3′UTR of LAMA4 mRNA (Fig. 5d). Also, miR-30e-3p mimic and LAMA4 overexpression plasmid vectors were separately transfected or co-transfected into SKOV3 and OVCAR3 cells. As shown in Fig. 5e, the level of miR-30e-3p increased by almost threefold when miR-30e-3p mimic was transfected into SKOV3 and OVCAR3 cells. The western blot results, illustrated in Fig. 5f, showed that the levels of endogenous and exogenous LAMA4 were reduced by more than 50% with miR-30e-3p mimic transfection. Interestingly, the co-transfection of LAMA4 overexpression plasmids with miR-30e-3p did not completely compromise the effects of miR-30e-3p on LAMA4 protein expression. The CCK-8 assay results in Fig. 5g showed that forced LAMA4 overexpression significantly reduced the proliferation of both cell lines by approximately a third, whereas the co-transfection of miR-30e-3p mimic with LAMA4 overexpression plasmids markedly enhanced cell proliferation compared with the LAMA4 overexpression group at 48 h and 72 h. The proliferation nonetheless was significantly weaker than that of the control group. Meanwhile, the results of the transwell invasion assays in Fig. 5h displayed that the number of invading cells in the co-transfection group was significantly more than that in LAMA4 overexpression group but significantly less than that in the control group in both SKOV3 cells and OVCAR3 cells. These findings proved that miR-30e-3p played a crucial role in OC development by targeting LAMA4 in vitro.

### MEG3 impaired cell proliferation and up-regulated the LAMA4 expression by sponging miR-30e-3p in OC cells

**Fig. 6:**
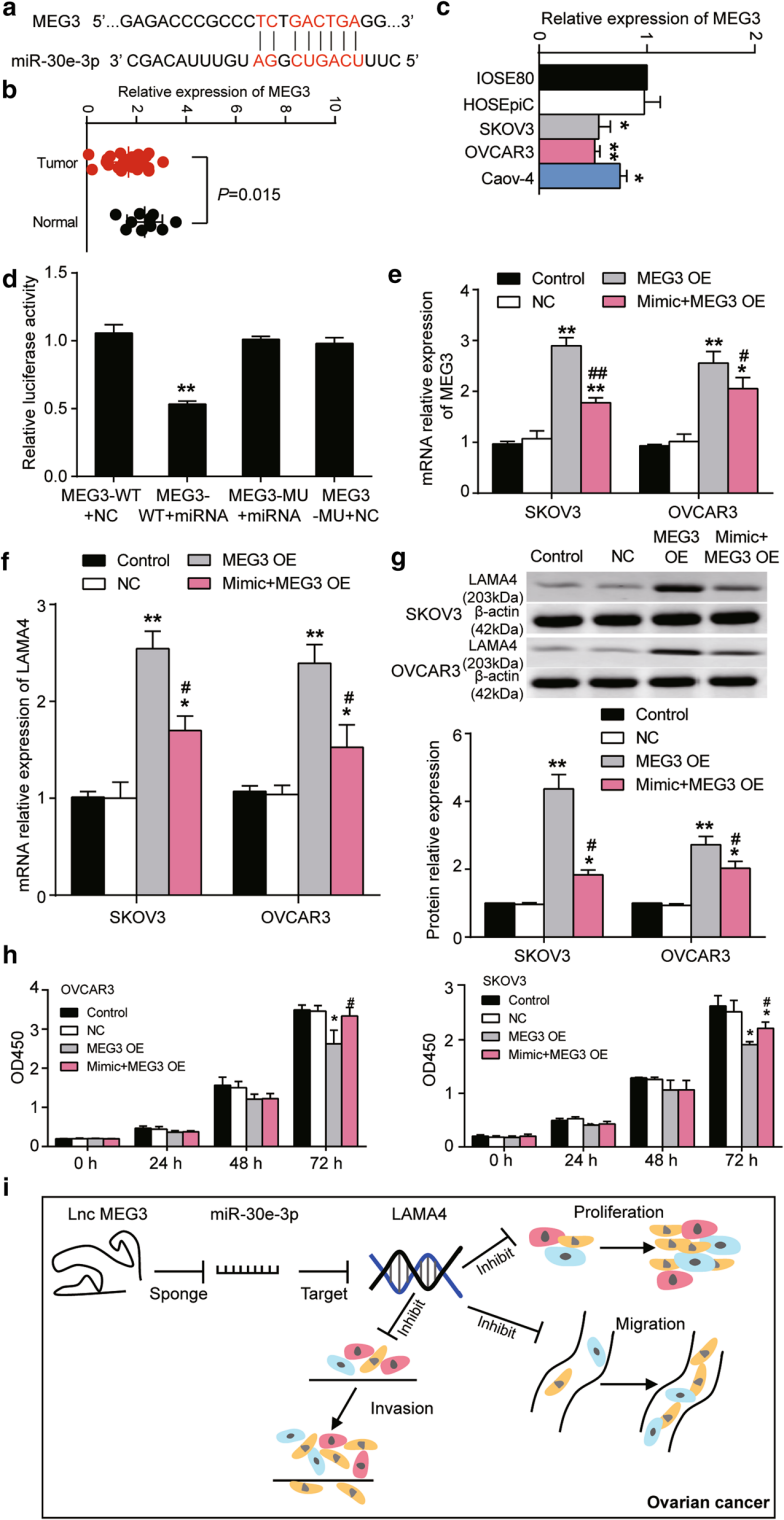
MEG3 promoted the expression of LAMA4 via sponging miR-30e-3p, and reversed the promoting effect of miR-30e-3p on the proliferation of SKOV3 and OVCAR3 cells. **a** The binding scheme between MEG3 and miR-30e-3p. The binding sequences were predicted using LncBase Experimental V.2. **b** The relative expression of MEG3 in healthy and cancerous ovarian tissues. **c** The relative expression of MEG3 in IOSE80 and HOSEpiC cell lines contrasting to SKOV3, OVCAR3 and Caov-4 cell lines. *P < 0.05, **P < 0.01 vs. IOSE80. **d** The relative luciferase activity decreased after the 293T cells were co-transfected with miR-30e-3p mimic and pGL3-MEG3-Wt. MEG3-WT + NC, the cells were transfected with pGL3-MEG3-Wt. MEG3-WT + miRNA, the cells were co-transfected with pGL3-MEG3-Wt and miR-30e-3p mimic. MEG3-MU + miRNA, the cells were co-transfected with pGL3-MEG3-Mut and miR-30e-3p mimic. MEG3-MU + NC, the cells were transfected with pGL3-MEG3-Mut. **P < 0.01 vs. MEG3-WT + NC. **e** MEG3 over-expression plasmid transfection successfully resulted in the significant upregulation of MEG3 in both cell lines, which was reversed by miR-30e-3p mimc. MEG3 over-expression plasmid was constructed using the pcDNA3.1 vector. **f**, **g** The mRNA and protein expression of LAMA4 were respectively detected using qRT-PCR and western-blot assay in SKOV3 and OVCAR3 cells with MEG3 over-expression alone or MEG3 over-expression with miR-30e-3p mimic. **h** The proliferation of SKOV3 and OVCAR3 cells with MEG3 over-expression or MEG3 over-expression with miR-30e-3p mimic using CCK8 assay. *OD450* optical density at 450 nm. **i** A scheme showing the mechanism involving MEG3, miR-30e-3p and LAMA4 in OC. Control group is the blank group. NC, the cells were transfected with empty plasmids. MEG3 OE, the cells were transfected with MEG3 over-expression plasmids. Mimic + MEG3 OE, the cells were co-transfected with miR-30e-3p mimic and MEG3 over-expression plasmids. *P < 0.05, **P < 0.01 vs. Control. ^#^P < 0.05, ^##^P < 0.01 vs. MEG3 OE group

LncBase Experimental V.2 was used to predict the potential binding site of miR-30e-3p on MEG3, and the binding sequences are illustrated in Fig. 6a. MEG3 expression was detected in the collected clinical tissue samples and cell lines. The results showed that MEG3 was significantly downregulated in OC tissues and cell lines. The expression of MEG3 in OC tissues was 27.9% lower than in healthy tissues (Fig. 6b). The expression of MEG3 in HOSEpiC cell line did not differ from that in IOSE80 cell line. The expression of MEG3 in SKOV3 and OVCAR3 cell line was approximately half of that in IOSE80 cell line. The expression of MEG3 in Caov4 cell line was 0.75 of that in IOSE80 cell line (Fig. 6c). A luciferase reporter assay was performed in the 293T cell line to validate the regulatory binding relationship between MEG3 and miR-30e-3p. Compared to other groups, a significant decrease of approximately 50% in the relative luciferase activity of MEG3-WT + miRNA cells was observed (Fig. 6d). MEG3 overexpression was successful but compromised by the co-transfection of miR-30e-3p mimic. Exogenous MEG3 overexpression resulted in up to threefold increase of MEG3, which was partially reversed by the introduction of miR-30e-3p mimic (Fig. 6e). The results proved that miR-30e-3p was regulatorily associated with MEG3. Additionally, the forced upregulation of MEG3 by transfecting MEG3 overexpression plasmids into cells resulted in significant upregulation of LAMA4 mRNA by about 2.5-fold (Fig. 6f), and LAMA4 protein by over fourfold in SKOV3 cell line and by approximately threefold in the OVCAR3 cell line (Fig. 6g). This upregulation was partly reversed by the co-transfection of miR-30e-3p mimic. Compared with the control group, the proliferation in MEG3 overexpression group was significantly suppressed in both cell lines at 72 h. Compared with the MEG3 overexpression group, the ability of cell proliferation in the co-transfection group was enhanced in both SKOV3 and OVCAR3 cells at 72 h after transfection (Fig. 6h). These results indicated that the MEG3 overexpression could suppress the proliferation of SKOV3 and OVCAR3 cells through sponging miR-30e-3p, thereby enhancing the expression of LAMA4. The mechanism is illustrated in Fig. 6i.

## Discussion

According to global cancer statistics, OC has contributed to the mortality of almost 184,799 women, and patients suffering from OC mainly died at the terminal stage of this cancer. Hence, understanding the underlying mechanism of OC is essential to OC treatments. In this study, bioinformatics analysis revealed that LAMA4 participated actively in OC metastasis. We found that LAMA4 overexpression suppressed the proliferation, migration, invasion, and colony formation of OC cell lines in vitro. Our results also proved that lncMEG3 enhanced LAMA4 expression by sponging miR-30e-3p, which was crucial in suppressing OC development.

In 1996, Richards et al. [20] found that LAMA4 was widely expressed in lung, skin, and pancreas. In recent years, some researchers paid close attention to this gene and explored the effect of LAMA4 on cancers. In human hepatocellular carcinoma, LAMA4 with high expression was observed, and findings indicated that LAMA4 upregulation had a strong correlation with hepatocellular tumor invasion [17]. Li et al. also found that LAMA4 overexpression induced cell migration in renal cell carcinoma via the ILK/FAK/ERK pathway. It seems that LAMA4 may be an oncogene for cancers. However, GEPIA, as well as our results in vivo and in vitro, showed that LAMA4 expression was markedly reduced in OC. Although research that focused on the effect of LAMA4 on OC was limited in scope, Yamamoto et al. detected the LAMA4 expression in OC by qRT-qPCR and observed that LAMA4 was significantly downregulated in OC ascites compared with healthy peritoneal fluids. In our study, we proved that LAMA4 overexpression repressed the proliferation, migration, and invasion of OC cells. In short, we indicated that LAMA4 was a tumor suppressor gene that inhibited OC progression.

To understand how LAMA4 influenced OC growth, miRNAs as the master regulators of gene expression were first considered. These small noncoding RNAs could reduce the expressions of oncogenes or tumor suppressor genes in participating in tumor development [21–23]. In renal cell carcinoma, miR-30e-3p was proved to be reduced. What’s more, miR-30e-3p overexpression inhibited cell invasion and metastasis by targeting Snail1 [24]. However, Lee et al. measured the expressions of nine miRNAs in 171 OC tissue specimens using Taqman-based RT-PCR and proved the high level of miR-30e-3p in 109 ovarian carcinoma tissues compared with the 22 normal ovarian tissues. Similar to Lee et al., our result also indicated that miR-30e-3p expression was upregulated in OC tissues and cells. We also proved that by targeting LAMA4, the upregulation of miR-30e-3p alleviated the anti-proliferation and invasion effects of LAMA4 over-expression.

Plenty of evidence has even revealed that the abnormal expression of lncRNAs exerts their effects on the progression of cancers by sponging miRNAs to regulate gene expression [25–28]. Lnc MEG3 was proved to be downregulated in OC tissues, and the mRNA expression of MEG3 was not measured in OC cells due to the hypermethylation of the MEG3 promoter [29]. In epithelial ovarian cancer (EOC), the MEG3 overexpression inhibited tumorigenesis by increasing early-stage cell apoptosis in a xenograft mouse model. Zhang et al. [30] found that curcumin resulted in the demethylation of MEG3 promoter to repress cell survival in OC cells, a process that could reduce drug resistance effectively. Consistent with these results, we observed that MEG3 expression was reduced in OC tissues and cells. More importantly, we observed that upregulated MEG3 contributed to the expression of LAMA4 by sponging miR-30e-3p, and the proliferation potential of OC cells was impaired.

Certain limitations existed in the present study. Firstly, an in vivo assay was not conducted to confirm the in vitro effects of the three molecules of interest. In our future work, we would consider this limitation and validate the roles of MEG3, miR-303-3p and LAMA4 in animal models. Another limitation was that we did not include an essential signaling cascade in our work. A signaling study only remained at the bioinformatics analysis level. Further research is also required to comprehend how the interaction between the three molecules influences signaling cascades within OC cells.

## Conclusions

In this study, we proved that forced LAMA4 overexpression could effectively impair the abilities of cell proliferation, migration, and invasion in OC cells. Our findings revealed that lncMEG3 regulated LAMA4 expression by sponging miR-30e-3p, thus resulting in the partial suppression of OC development. Even though the mechanism of LAMA4 requires further investigation, our results may provide a novel strategy for diagnosing and treating OC.

## Data Availability

The datasets used and analysed during the current study are available from the corresponding author on reasonable request.

## Abbreviations

CCK-8: Cell Counting Kit-8
EOC: Epithelial ovarian cancer
FBS: Fetal bovine serum
GO: Gene Ontology
LAMA4: Laminin alpha4
LncRNAs: Long non-coding RNAs
NC: Negative control
OC: Ovarian cancer
